# A Molecular Surveillance System for Global Patterns of Drug Resistance in Imported Malaria

**DOI:** 10.3201/eid0901.020121

**Published:** 2003-01

**Authors:** Annie-Claude Labbé, Samir Patel, Ian Crandall, Kevin C. Kain

**Affiliations:** * Toronto General Hospital, University of Toronto, Canada

**Keywords:** Malaria, drug-resistance, molecular surveillance, *Plasmodium falciparum*, PCR, travelers, research

## Abstract

Analysis of imported malaria in travelers may represent a novel surveillance system for drug-resistant malaria. We analyzed consecutive falciparum malaria isolates from Canadian travelers from 1994 to 2000, for polymorphisms in *pfcrt,* *dhfr,* and *dhps* linked to chloroquine and pyrimethamine/sulfadoxine resistance. Forty percent of isolates possessed the K76 *pfcrt* allele, suggesting that many imported falciparum infections are still responsive to chloroquine. Travelers who had recently taken chloroquine had a significantly increased risk of harboring isolates with *pfcrt* resistance alleles (odds ratio = 4.47; p=0.03). The presence of two or more mutations in *dhfr* or *dhps* was found in 64.8% (95% confidence interval [CI] 54.6 to 73.9) and in 30.4% (95% CI 21.7 to 40.3) of isolates, respectively, and increased significantly over the course of the study. These molecular markers indicate that pyrimethamine/sulfadoxine resistance is increasing and is now too high to rely on this drug as a routine therapeutic agent to treat malaria in travelers.

Drug-resistant malaria is increasing, and novel strategies to monitor for resistance are needed. Over 50 million persons from the industrialized world visit malaria-endemic countries annually, and record numbers of imported malaria cases are being reported in North America and Europe (*1*). The first well-documented cases of chloroquine-resistant and sulfadoxine-pyrimethamine (SP)–resistant *Plasmodium falciparum* malaria were identified in tourists visiting East Africa in the late 1970s and early 1980s, which suggests that travelers may represent an important sentinel population to monitor for drug-resistant malaria (*2*,*3*). Although assessing travelers for malaria treatment and prophylaxis failures may be an effective strategy for detecting emerging drug resistance, traditional methods of detecting resistance, including in vivo treatment trials and in vitro drug susceptibility testing, are time- and labor-intensive and are not well suited to large-scale surveillance of travelers (*4*).

Molecular methods that detect genetic markers of drug resistance in parasites are potentially powerful tools to detect and track drug-resistant malaria. The molecular basis of resistance to antifolate drugs such as SP has been well characterized. High-level pyrimethamine resistance results from the accumulation of mutations in the *dhfr* gene, principally at codons 108, 59, and 51 (*5*,*6*). Similarly, point mutations in *dhps* have been associated with decreased susceptibility to sulfadoxine in vitro (*7*). Chloroquine resistance has been linked to mutations in two genes, *pfmdr1* and *pfcrt*, that encode the digestive vacuole transmembrane proteins Pgh1 and PfCRT, respectively (*8*–*13*). Transfection studies with *pfmdr1* suggest that mutations in Pgh1 may modulate the chloroquine resistance phenotype in vitro; however, in vivo studies have shown an inconsistent association between mutations in Pgh1 and chloroquine resistance (*9*–*12*). More recently, a series of point mutations in *pfcrt* have been associated with chloroquine resistance (*13*). One mutation at position 76 (K76T) was present in all in vitro resistant parasites and has been proposed as a molecular marker for surveillance of chloroquine-resistant falciparum malaria, particularly in nonimmune populations such as travelers (*10*,*13*).

The objectives of this study were to establish a molecular surveillance system for imported malaria, to determine and track the prevalence of putative molecular markers of drug resistance, and to examine risk factors for infection with isolates bearing resistance markers.

## Materials and Methods

From January 1, 1994, to June 30, 2000, patients seen at the Toronto General Hospital or the Hospital for Sick Children in Toronto, Canada, with microscopically confirmed falciparum malaria were enrolled. Patient interviews were conducted or medical charts were reviewed for potential risk factors for infection and drug resistance by using a standardized data extraction form. This study was approved by the Institutional Review Boards of the Toronto General Hospital and the Hospital for Sick Children.

Falciparum isolates were characterized by polymerase chain reaction-restriction fragment length polymorphism analysis and sequencing for allelic variants of *pfcrt* and *pfmdr1*, *dhfr,* and *dhps* as described (*5*–*17*). Proportions were compared by using the chi-square test or Fisher exact test, as appropriate. The chi-square test for trend was used as required for variables that involved ordered categories. For the purpose of this analysis, we considered mixed isolates (e.g., isolates containing parasites with mutant and wild-type alleles) to be mutant ones and compared them against those possessing only wild-type alleles.

## Results

During the study period, 105 consecutive cases of imported falciparum malaria were recorded (69 males, 36 females; age range 1–70 years [mean 30.5]). The geographic regions in which these persons acquired their infections are shown in Table 1.

**Table 1:** Prevalence of molecular markers of drug resistance by region of malaria acquisition

	Area of endemicity n (%)					Total
Genotypes	West Africa^a^	East Africa^b^	Central Africa^c^	Southern Africa^d^	Other^e^	n (%; 95%CI)^f^
*pfmdr1*						
N86 (wild)	40 (59.7)	5 (29.4)	1 (20.0)	1 (33.3)	6 (75.0)	53 (53.0; 42.8 to 63.1)
86Y (mutant)	27 (40.3)	12 (70.6)	4 (80.0)	2 (66.7)	2 (25.0)	47 (47.0; 36.9 to 57.2)
*pfcrt*						
K76 (wild)	32 (49.2)	3 (17.6)	3 (60.0)	0	1 (11.1)	39 (39.8; 30.0 to 50.2)
76T (mutant)	33 (50.8)	14 (82.4)	2 (40.0)	2 (100)	8 (88.9)	59 (60.2; 49.8 to 70.0)
*dhfr* ^g^						
Wild-type	17 (25.0)	3 (17.6)	3 (60.0)	1 (33.3)	2 (22.2)	26 (25.5; 17.4 to 35.1)
Single mutants	6 (8.8)	0	0	0	3 (33.3)	9 (8.8; 4.1 to 16.1)
Double mutants	25 (36.8)	8 (47.1)	1 (20.0)	2 (66.7)	3 (33.3)	39 (38.2; 28.8 to 48.4)
Triple mutants	20 (29.4)	6 (35.3)	1 (20.0)	0	1 (11.1)	28 (27.5; 19.1 to 37.2)
*dhps* ^h^						
Wild-type	3 (4.4)	7 (41.2)	0	2 (66.7)	8 (88.9)	20 (19.6; 12.4 to 28.6)
Single mutants	40 (58.8)	5 (29.4)	5 (100)	1 (33.3)	0	51 (50.0; 40.0 to 60.1)
Double mutants	21 (30.9)	5 (29.4)	0	0	0	26 (25.5; 17.4 to 35.1)
Triple mutants	4 (5.9)	0	0	0	1 (11.1)	5 (4.9; 1.6 to 11.1)
No. of infected patients	71 (67.6)	17 (16.2)	5 (4.8)	3 (2.9)	9 (8.6)	105

^a^Two patients had visited more than one country: Ghana (45 patients), Nigeria (21), The Gambia (2 patients), Sierra Leone (3 patients), Burkina Faso (1 patient), Mali (1 patient), and Guinea (1 patient).

^b^Three patients had visited more than one country: Kenya (9 patients), Uganda (6 patients), Tanzania (3 patients), Rwanda (1 patient), and Burundi (1 patient).

^c^Central African Republic (2 patients), Congo (2 patients), and Cameroon (1 patient)

^d^Angola (2 patients) and Madagascar (1 patient).

^e^India (5 patients), Malaysia (1 patient), Bali/New Guinea (1 patient), Brazil (1 patient), and Haiti (1 patient).

^f^ CI, confidence interval.

^g^*dhfr*: Wild-type: parasites with A16 / C50 / N51 / C59 / S108 / I164 (n = 26). Single mutants: isolates with the S108N alone (n=9). Double mutants: parasites with mutations at codons N51I and S108N (n=11), C59R and S108N (n=27), or A16V and S108T (n=1). Triple mutants: parasites with the genotypes of N51I / C59R / S108N (n=27) or C50R / N51I / S108N (n=1). Of note, the falciparum isolate with the A16V/S108T mutations was acquired in 1996 by a 12-year-old in Ghana. Those mutations in *dhfr* were not accompanied by the mutant codon I164L, previously associated with pyrimethamine and cycloguanil resistance (*17*).

^h^*dhps*: Wild-type parasites: parasites with S436 / A437 / K540 / A581 / A613 (n=20). Single mutants: isolates with the S436A (n=19) or A437G (n=32) mutation alone. Double mutants: parasites with mutations at codons S436A and A437G (n=18), A437G and K540E (n=6), or S436F and A613S (n=2). Triple mutants: parasites with S436A / A437G / A613S (n = 3), S436A / A437G / A581G (n=1), or A437G / K540E / A581G (n=1). Note: Some isolates could not be amplified at all loci and account for occasional missing values.

The prevalence of mutations in chloroquine-resistance markers (*pfmdr1* and *pfcrt*) was significantly higher in isolates acquired in East Africa compared with West Africa (Table 1). Patients who acquired malaria infection in East Africa had a 4.5-fold higher risk of being infected by an isolate possessing the K76T mutation in *pfcrt* (odds ratio [OR] 4.53 [95% confidence interval (CI) 1.26 to 16.02]; p=0.03) than those visiting West Africa. The N86Y mutation in *pfmdr1* was also found more often in isolates acquired in East Africa than in those acquired in West Africa (OR 3.56 [95% CI 1.16 to 10.80]; p=0.03). A linear trend for increasing prevalence of N86Y mutant isolates was evident during the study period (OR per year =1.21; p=0.07) (Table 2). We also examined the association between past chloroquine exposure and the prevalence of chloroquine-resistance markers. Nineteen (18.1%) patients had used chloroquine for prophylaxis (n=13) or treatment (n=6) while abroad. These persons had a significantly increased risk of being infected by an isolate harboring the K76T mutation when compared with other travelers (OR=4.47 [95% CI 1.13 to 25.45]; p=0.03).

**Table 2:** Proportions of falciparum isolates with chloroquine- or sulfadoxine-pyrimethamine-associated resistance markers by year of acquisition^a^

Year
	Proportions of mutant isolates				Proportions of isolates with at least 2 mutant codons			
	*pfmdr1* (N86Y)^b^		*pfcrt* (K76T)		*dhfr* ^c^		*dhps* ^d^	
1994	42.9%	9/21	71.4%	15/21	45.5%	10/22	14.3%	3/21
1995	25.0%	2/8	55.6%	5/9	62.5%	5/8	33.3%	3/9
1996	31.6%	6/19	50.0%	9/18	52.4%	11/21	25.0%	5/20
1997	52.9%	9/17	66.7%	10/15	88.2%	15/17	35.3%	6/17
1998	64.3%	9/14	50.0%	7/14	85.7%	12/14	21.4%	3/14
1999	50.0%	7/14	64.3%	9/14	78.6%	11/14	57.1%	8/14
2000^e^	71.4%	5/7	57.1%	4/7	50.0%	3/6	42.9%	3/7
Total	47.0%	47/100	60.2%	59/98	65.7%	67/102	30.4%	31/102
(95% CI)	(36.9 to 57.2)		(49.8 to 70.0)		(54.6 to 3.9)		(21.7 to 40.3)	

^a^OR, odds ratio; CI, confidence interval.

^b^OR for a 1-unit increase in year = 1.21 (95% CI 0.99 to 1.49); p=0.07, chi-square test for trend.

^c^OR for a 1-unit increase in year = 1.28 (95% CI 1.0 to 1.59); p=0.02, chi-square test for trend.

^d^OR for a 1-unit increase in year = 1.29 (95% CI 1.03 to 1.61); p=0.03, chi-square test for trend.

^e^Data for year 2000 are from January 1 to June 30.

We grouped the parasite *dhfr* and *dhps* genotypes into four categories on the basis of the cumulative number of mutations that have been linked to escalating SP resistance (Table 1). More mutations in *dhps* were found in isolates from travelers returning from West Africa versus East Africa (p=0.001, Fisher exact test). We found that the proportion of isolates with at least two mutations increased during the study period in both *dhfr* (OR for a 1-unit increase in year = 1.28; p=0.02, chi-square test for trend) and *dhps* (OR=1.29; p=0.03) (Table 2).

## Discussion

In this study, we demonstrate “proof-of-principle” that a molecular surveillance strategy based on imported malaria in travelers can be used to detect and track drug-resistant malaria. Monitoring travelers for imported drug-resistant malaria is a surveillance strategy that offers several potential advantages. Recommendations regarding treatment regimens and chemoprophylaxis for travelers should ideally be made on the basis of the efficacy of these drugs in nonimmune travelers rather than on partially immune persons residing in malaria-endemic areas. However, to date there has been little information on the rates of drug resistance in cases of imported malaria. Using travelers as a sentinel system provides a mechanism to study large numbers of persons returning from diverse malaria-endemic areas. In contrast, traditional studies have often been based on relatively small numbers of persons residing in geographically restricted areas. Travelers are generally nonimmune, facilitating the interpretation of treatment and prophylaxis studies since outcome measures are not confounded by reinfections and by the varying degrees of immunity present in residents of malaria-endemic areas. Similarly, correlating the molecular mechanisms of drug resistance to treatment outcome in travelers may be more straightforward since these confounding variables can largely be excluded. Knowledge of the resistance genotypes of malaria parasites obtained from returning travelers can provide credible and complementary data for evidence-based recommendations for both chemoprophylaxis and therapy of malaria in travelers.

The high correlation between mutations in DHFR and DHPS and in vitro resistance to pyrimethamine and sulfadoxine, further supported by site-directed mutagenesis and transfection experiments, suggests that the epidemiology of antifolate resistance in *P. falciparum* can be monitored by molecular techniques (*5*–*7*,*14*–*17*). Furthermore, evidence exists for an association between a stepwise increase in the number of mutations in DHFR and DHPS and a corresponding increase in the level of clinical resistance to SP. In recent in vivo studies in partially immune persons in Cameroon and Kenya, multiple mutations in DHFR (e.g., triple mutation at codons 108, 59, and 51) were associated with early treatment failure, suggesting that these could be useful markers for predicting the in vivo efficacy of SP (*18*–*20*).

Using molecular markers of antifolate resistance, our study provides important data on the appropriateness of drugs such as SP that are currently recommended in North America and Europe for treatment or self-treatment of malaria in travelers. We observed that 75%, 66%, and 28% of consecutive imported isolates had at least one, two, and three mutations in DHFR, respectively. In DHPS, corresponding figures were 80%, 30%, and 5%. Furthermore, we found a linear trend for increasing prevalence mutations in *dhfr* and *dhps* during this study. These results suggest that antifolate resistance in imported falciparum malaria is now common and escalating over time. These observations question the rationale of continued recommendation of SP as either standby therapy or combination therapy with quinine for the treatment of *P. falciparum* malaria in travelers. However, some caution is needed in extrapolating our data to predict the in vivo efficacy of SP. Additional prospective in vivo studies, especially in the nonimmune host, are required to definitively link antifolate molecular markers with in vivo resistance (*18*–*20*).

We have also collected data on the occurrence of mutations associated with chloroquine resistance in consecutive imported falciparum isolates. The overall prevalence of the N86Y mutation in *pfmdr1* and K76T mutation in *pfcrt* was 47.0% and 60.2%, respectively. Recent in vivo studies have assessed the association between *pfcrt* mutations and chloroquine response and determined that the K76 allele correctly predicted successful outcome (*9*–*11*). A rapid assay to detect *pfcrt* K76T in travelers’ malaria may be useful, since the presence of the K76 allele would indicate the probable effectiveness of treatment with chloroquine alone. On the basis of these findings, we anticipate that at least 40% of our patients would have responded to chloroquine. However, in the absence of a rapid test, current recommendations from the World Health Organization and the Centers for Disease Control and Prevention must be applied, and *P. falciparum* infections acquired in areas of known chloroquine resistance should not be treated with chloroquine.

Chloroquine and SP resistance has been selected by drug pressure (*5*,*6*,*10*,*21*). In Mali, the K76T mutation in *pfcrt* was observed in persons with persistent or recurrent infection after chloroquine therapy, indicating selection for this mutation. Our study extends these observations to travelers; those who had taken chloroquine for prophylaxis or treatment had a 4.5-fold higher risk of being infected with an isolate carrying the K76T mutation. Although the number of isolates studied was relatively small, our results also indicate that the prevalence of genotypes associated with chloroquine resistance was significantly higher in isolates acquired in East Africa than in those acquired in West Africa. This observation is consistent with currently reported epidemiologic patterns (*22*). The distribution of chloroquine- and SP-resistant parasites and their degree of resistance are far from uniform, and regular assessment of the therapeutic efficacy of chloroquine and SP, such as obtained with World Health Organization in vivo studies, is required. Studies such as ours, using travelers as sentinels, can contribute in a novel and complementary way to the continuous monitoring and tracking of geographic drug-resistance patterns. A network of digitally linked sites in the developed world that are performing these analyses in cases of imported malaria could provide global and timely monitoring.

In summary, our study demonstrates that a molecular surveillance strategy based on imported malaria in travelers can be used to detect and track patterns of drug-resistant malaria. Given the high prevalence of observed mutations in *dhfr* and *dhps*, our data provide evidence that raises questions about the rationale of continued use of SP to treat falciparum malaria in returned travelers. Our data also indicate that a considerable proportion of imported falciparum infections are still responsive to chloroquine.
